# The Potential Value of Texture Analysis Based on Dynamic Contrast-Enhanced MR Images in the Grading of Breast Phyllode Tumors

**DOI:** 10.3389/fonc.2021.745242

**Published:** 2021-11-10

**Authors:** Xiaoguang Li, Hong Guo, Chao Cong, Huan Liu, Chunlai Zhang, Xiangguo Luo, Peng Zhong, Hang Shi, Jingqin Fang, Yi Wang

**Affiliations:** ^1^ Department of Radiology, Daping Hospital, Army Medical University, Chongqing, China; ^2^ GE Healthcare, Shanghai, China; ^3^ Department of Pathology, Daping Hospital, Army Medical University, Chongqing, China; ^4^ Department of Information, Daping Hospital, Army Medical University, Chongqing, China

**Keywords:** breast, phyllodes tumors, magnetic resonance imaging, texture analysis, differential diagnosis

## Abstract

**Purpose:**

To explore the value of texture analysis (TA) based on dynamic contrast-enhanced MR (DCE-MR) images in the differential diagnosis of benign phyllode tumors (BPTs) and borderline/malignant phyllode tumors (BMPTs).

**Methods:**

A total of 47 patients with histologically proven phyllode tumors (PTs) from November 2012 to March 2020, including 26 benign BPTs and 21 BMPTs, were enrolled in this retrospective study. The whole-tumor texture features based on DCE-MR images were calculated, and conventional imaging findings were evaluated according to the Breast Imaging Reporting and Data System (BI-RADS). The differences in the texture features and imaging findings between BPTs and BMPTs were compared; the variates with statistical significance were entered into logistic regression analysis. The receiver operating characteristic (ROC) curve was used to assess the diagnostic performance of models from image-based analysis, TA, and the combination of these two approaches.

**Results:**

Regarding texture features, three features of the histogram, two features of the gray-level co-occurrence matrix (GLCM), and three features of the run-length matrix (RLM) showed significant differences between the two groups (all p < 0.05). Regarding imaging findings, however, only cystic wall morphology showed significant differences between the two groups (p = 0.014). The areas under the ROC curve (AUCs) of image-based analysis, TA, and the combination of these two approaches were 0.687 (95% CI, 0.518–0.825, p = 0.014), 0.886 (95% CI, 0.760–0.960, p < 0.0001), and 0.894 (95% CI, 0.754–0.970, p < 0.0001), respectively.

**Conclusion:**

TA based on DCE-MR images has potential in differentiating BPTs and BMPTs.

## Introduction

Phyllodes tumors (PTs) of the breast are rare fibroepithelial neoplasms, accounting for 0.3% to 1% of all primary breast tumors. PTs are classified as benign, borderline, and malignant, according to the latest edition of the World Health Organization (WHO) classification of the breast, which is based on the semiquantitative evaluation of key histological features, such as stromal cellularity, stromal atypia, stromal mitosis, stromal overgrowth, and tumor margin. Surgery is an essential means to treat PTs, and different surgical methods are commonly selected according to histologic grade. Generally, local excision is applied for BPTs, and wide excision or mastectomy is used for BMPTs (1). Therefore, the preoperative differentiation between benign and malignant PTs would be significant for surgery planning. Although a fine-needle biopsy is sometimes helpful in determining the preoperative diagnosis of PTs, it is insufficient for PT grading because of potential inadequate cytologic samples resulting from the heterogeneous nature of PTs (2).

Magnetic resonance imaging (MRI) is a well-established method in breast imaging, with various clinical applications, including the noninvasive differentiation between benign and malignant breast lesions, preoperative staging, detection of recurrence, and the evaluation of prognosis (3, 4). At present, dynamic contrast-enhanced MRI (DCE-MRI) is the most sensitive imaging technique for breast cancer diagnosis and provides excellent morphological and, to some extent, also functional information (4). However, breast MRI still has limitations in the differentiation between benign and malignant PTs to date. Firstly, MRI morphology alone does not differentiate benign from malignant PTs (5). Secondly, many studies have reported that noncontrast MRI has little significance in the differentiation of benign and malignant PTs (5, 6). Even functional imaging parameters, such as the ADC value, still have contradictions in different studies (7, 8). Finally, PTs could demonstrate significant enhancement on DCE-MRI, regardless of histological type, which may be related to angiogenesis factors that promote the growth of matrix and epithelial components (9). Therefore, it would be valuable to find a new way to improve the diagnostic performance of MR images in differentiating BPTs from BMPTs.

Recently, with the rapid development of digital image processing, texture analysis (TA) has become an important quantitative method for medical image analysis. Compared with the overall or qualitative reports of tumor appearance, TA can provide an accurate local description of tumor complexity, heterogeneity, and dynamic behavior on medical images (10). Many previous studies have shown that TA of DCE-MRI can provide an opportunity to promote clinical decision-making in terms of low-cost and noninvasive evaluation of tumors, such as in histopathologic and molecular subtype classification of breast cancer (11), tumor prognosis (12), and treatment response prediction (13, 14). However, few studies have shown the role of texture features based on DCE-MR images in PT grading (15). The purpose of this study, therefore, was to explore the value of TA based on DCE-MR images in distinguishing BPTs from BMPTs.

## Materials and Methods

### Patients

This study was approved by the institutional ethics committee of our hospital [Ratification NO: 2019(160)]. The need for informed consent was waived by the institutional review board (IRB) due to the nature of this retrospective study. The DCE-MRI data of 55 patients with histopathological confirmed PTs from November 2012 to March 2021 were reviewed, and 47 eligible patients were enrolled in this study finally. The exclusion criteria included the following: 1) low-quality images cannot be used for subsequent analysis (n = 3); 2) a history of breast implants in one or both sides (n = 2); and 3) MRI scanning after surgery, chemotherapy, or radiotherapy (n = 3). All the patients were female and between the ages of 16 and 71 years (mean 44.30 ± 10.26 years). Each patient had only 1 lesion in the unilateral breast, 20 lesions in the left, and 27 lesions in the right. Of the 47 PT cases, 26 were benign, 18 were borderline, and 3 were malignant.

### Imaging Protocol

MRI was performed using a 1.5-T scanner (MAGNETOM Aera, Siemens Healthcare, Erlangen, Germany) with a dedicated eight-channel breast coil. The MRI protocol included axial turbo inversion recovery magnitude (TIRM) T2WI with fat saturation (T2WI_FS_), axial FL3D-T1WI with nonfat saturation, DWI, and DCE-MRI based on the FL3D sequence. The detailed scan parameters were as follows: T2WI_FS_ (TR 5,600 ms, TE 57 ms, FOV 340 mm×340 mm); T1-FL3D (TR 8.6 ms, TE 4.7 ms, FOV 360 mm×360 mm; DWI (TR 6,300 ms, TE 68 ms, FOV 340 mm × 340 mm b=0, 50, 600, 1000 s/mm^2^); and DCE-MRI (TR 4.62 ms, TE 1.75 ms, FOV 360 mm×360 mm). After a 90-s scan, the dynamic contrast-enhanced scan was performed. The contrast agent Gd-DTPA (Magnevist, Bayer Healthcare, Berlin, Germany) was injected into the elbow vein by a high-pressure syringe at a dose of 0.1 mmol/kg and a flow rate of 2.0 ml/s. Subsequently, seven phases were continuously collected without intervals. Each scanning duration was approximately 60.01 s, the layer thickness was 3 mm, and the total time was 7 min. After contrast agent injection, 15 ml of normal saline was injected at the same flow rate.

### Imaging Analysis

MR images of all patients were independently reviewed by two senior radiologists (CZ and X-Luo with 10 and 15 years of experience in breast imaging, respectively) blinded to the histopathological results, and the imaging findings were evaluated according to the BI-RADS MRI (16). The following descriptors were recorded: the maximum diameter, shape (round, oval, or irregular), margin (circumscribed or irregular), T2WI_FS_ signal, hyperintense on T2WI_FS_, hyperintense on T1WI, lobulation (absence or presence), cystic component (absence or presence), and if present, the wall of the cystic component (regular or irregular), internal enhancement characteristics (heterogeneous or homogeneous), and time signal intensity curve (TIC) patterns (type I, persistent pattern, the signal intensity rose continuously during the dynamic observation; type II, plateau pattern, the signal intensity was gradually increased at an early stage and then maintained at a platform level; type III, washout pattern, the signal intensity was increased rapidly at an early stage and then decreased rapidly) (17). All imaging findings were determined by consensus.

### Texture Analysis

MaZda software (version 4.7, The Technical University of Lodz, Institute of Electronics, http://www.eletel.p.lodz.pl/mazda/) was used for the TA. Based on our previous study (18), DCE-MR images at phase VII were selected for texture analysis, which showed the best contrast enhancement of PTs. To obtain the reproducible and dependable results for signal intensity measurement, the VOI (volume of interest) of each tumor, which encompassed the whole lesion on each consecutive slice, was manually delineated respectively by the above radiologists. For every VOI, gray-level normalization was performed using the limitation of dynamics μ ± 3σ (μ is the gray-level mean; σ is the gray-level standard deviation) to minimize the influence of contrast and brightness variations (19). Texture features derived from the gray-level histogram (HIS), the gray-level co-occurrence matrix (GLCM), the gradient matrix (GrM), and the run-length matrix (RLM) were calculated for the VOIs. The numbers of calculated features per feature class are as follows: HIS, n=9; GLCM, n=275; RLM, n=25; GRM n=5; (total number of features per lesion, n= 314). HIS features are calculated based on pixel intensity, regardless of the spatial relationships between pixels in the image (20). GLCM features are calculated based on how often pairs of pixels/voxels with specific values, which could provide information on lesion heterogeneity (21). GrM features are calculated for direction changes in gray-level intensity and represent the image intensity distribution (20). RLM features are calculated for five directions (Z-axis, horizontal, vertical, 45 degrees, and 135 degrees) and represent the number of times there is a run of pixels having a certain gray level (22, 23). The categories of the texture features are listed in **Table 1**. They can be accessed at (http://www.eletel.p.lodz.pl/programy/mazda/download/mazda_manual.pdf).

**Table 1:** List of texture features in the MaZda software.

Category	Features
Histogram (n = 9)	Mean (histogram’s mean); variance (histogram’s variance); skewness (histogram’s skewness); kurtosis (histogram’s kurtosis); Perc.01% (1% percentile); Perc.10% (10% percentile); Perc.50% (50% percentile); Perc.90% (90% percentile); Perc.99% (99% percentile)
Gray-level co-occurrence matrix (n = 275)	AngScMom, Contrast, Correlat, SumOfSqs, InvDfMom, SumAverg, SumVarnc, SumEntrp, Entropy, DifVarnc, DifEntrp. Features are computed for five between-pixels distances (1, 2, 3, 4, 5) and for four various directions (horizontal, vertical, 45 degrees, 135 degrees)
Run-length matrix (n = 25)	RLNonUni, GLevNonU, LngREmph, ShrtREmp, Fraction. Features are computed for 5 various directions (Z-axis, horizontal, vertical, 45 degrees, 135 degrees)
Absolute gradient (n = 5)	GrMean, GrVariance, GrSkewness, GrKurtosis, GrNonZeros (percentage of pixels with non-zero gradient)

AngScMom, angular second moment; Correlat, correlation; DifEntrp, difference entropy; DifVarnc, difference variance; GLevNonU, gray-level non-uniformity; GrKurtosis, absolute gradient kurtosis; GrMean, absolute gradient mean; GrNonZeros, percentage of pixels with nonzero gradient; GrSkewness, absolute gradient skewness; GrVariance, absolute gradient variance; InvDfMom, inverse difference moment; LngREmph, long run emphasis; n = total number of texture features of each category extracted from MaZda; RLNonUni, run length non-uniformity; ShrtREmp, short run emphasis; SumAverg, sum average; SumEntrp, sum entropy; SumOfSqs, sum of squares; SumVarnc, sum variance.

### Statistical Analysis

**Figure 1:**
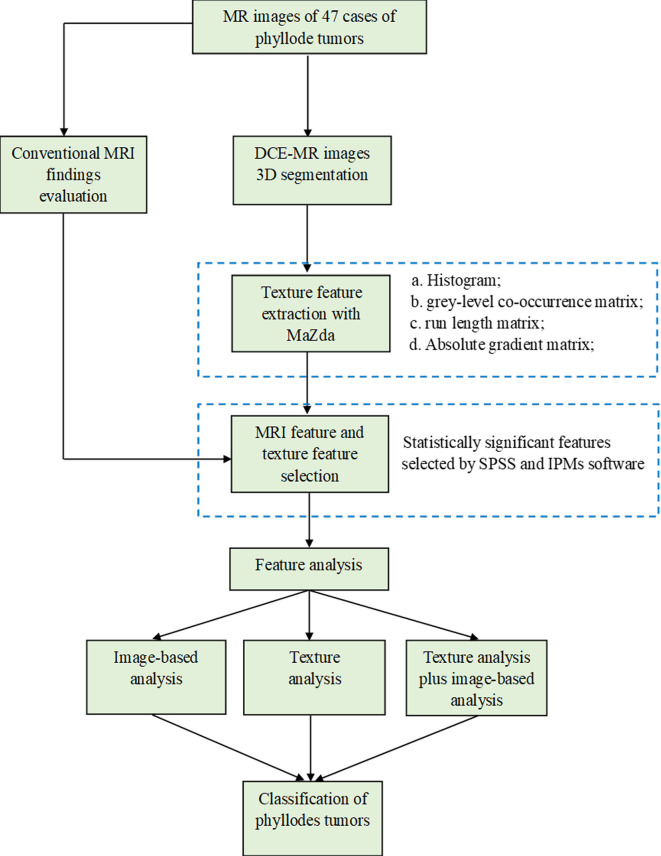
The workflow chart of this study.

Statistical analysis was performed using IBM SPSS version 21.0 (IBM Corporation, New York). With regard to the reproducibility of volumetric and texture analysis, interobserver reliability was assessed by intraclass correlation coefficient (ICC) test (0.000–0.200, poor; 0.201–0.400, fair; 0.401–0.600, moderate; 0.601–0.800, good; and 0.801–1.000, excellent). The Kolmogorov–Smirnov and Levene tests were used to determine the normality and homogeneity of the variance, respectively, for all measurement data. Intergroup comparisons were performed with independent sample t-tests and Mann–Whitney U tests for data with normal and abnormal distribution, respectively (24). Quantitative data with a normal distribution are expressed as the means ± standard deviations (SDs), while quantitative data with a skewed distribution are presented as median (quartile 1, quartile 3). Categorical data were shown as a percentage and were analyzed using the chi-square test or Fisher’s exact test. To evaluate the effect of conventional MRI findings on tumor classification, we include variables with a value of p < 0.20 for multivariate logistic regression. A p-value < 0.05 was considered to be statistically significant. In terms of feature selection, we applied the Institute of Precision Medicine Statistics (IPMs, version 2.0, GE Healthcare). Before feature selection, all parameters are processed by the standardization function of IPMs software to reduce differences in dimensions. The specific steps of feature selection and model establishment are as follows: Firstly, the variance threshold method was used to reduce the redundant features. The threshold value was 0.8; thus, the eigenvalues of the variance smaller than 0.8 were removed (25). Secondly, the univariate analysis was adopted to obtain features with statistically significant differences (p < 0.05) between BPT and BMPT groups. Thirdly, the univariate logistic regression analysis was used to retain the variables with statistical differences (p < 0.05). Finally, the promising features were fed into multivariate logistic regression analysis with a backward stepwise selection procedure for tumor classification. A combined model integrating promising imaging findings and texture features was also established. The goodness-of-fit of the logistic regression model was evaluated using the Hosmer–Lemeshow test (26). The diagnostic efficacy of these models based on image-based analysis, TA, and the combination of the two approaches was measured by the area under the curve (AUC) of ROC curves. The Delong test was adopted to compare AUCs. A p-value < 0.05 was considered to be statistically significant. A workflow chart of this study is illustrated in **Figure 1**.

## Results

### Comparison of Texture Features Between BPTs and BMPTs

**Figure 2:**
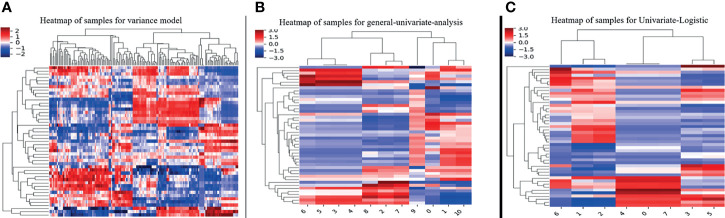
The steps of texture features reduction and selection by the method of **(A)** variance, **(B)** univariate analysis, and **(C)** univariate logistic regression.

The interobserver reproducibility of texture features extraction was good, with ICC values ranging from 0.71 to 0.98. In this study, 314 texture features were extracted from the DCE-MR images of each lesion (**Table 1**). A total of 263 nonsignificant features were first eliminated using variance analysis with the threshold value of 0.8 (**Figure 2A**). After removing the redundant features using univariate analysis (**Figure 2B**), a total of 11 significant features remained. Through univariate logistic regression analysis (**Figure 2C**), eight features with statistical differences were retained for further multivariate logistic regression analysis. For the HIS features, the Perc.90% (percentile 90%), mean, and variance in BMPTs were significantly lower than those in BPTs (p = 0.001, 0.003, and 0.004, respectively). For the GLCM features, the S(0,0,1)AngScMom and S(1,0,0)AngScMom in BMPTs were significantly lower than those in BPTs (p = 0.019 and 0.029, respectively). However, for the RLM features, the Z_GLevNonU, 45dgr_GLevNonU, and 135dr_GLevNonU (gray-level nonuniformity in Z-axis, 45, and 135 degree directions, respectively) in BMPTs were significantly higher (p = 0.039, 0.037, and 0.037, respectively) (**Table 2**).

**Table 2:** Comparisons of texture features from DCE-MR images between BPT and BMPT groups.

Variable	BPT (n = 26)	BMPT (n = 21)	F-value	p value
RLM				
Z_GLevNonU	370.02 (206.79, 1059.87)	1204.56 (489.95, 3072.55)	-2.054	0.039
135dr_GLevNonU	367.10 (207.28, 1061.48)	1236.88 (486.62, 3166.87)	-2.076	0.037
45dgr_GLevNonU	370.81 (203.73, 1066.45)	1224.05 (487.62, 3176.61)	-2.054	0.037
GLCM				
S (0,0,1) AngScMom	36.68 (25.39, 57.51)	24.99 (19.28, 42.06)	-2.054	0.019
S (1,0,0) AngScMom	42.01 (26.17, 60.59)	29.41 (22.36, 43.30)	-1.883	0.029
Histogram				
Perc.90%	43.17 (37.80, 52.05)	25.00 (15.70, 40.00)	3.338	0.001
Variance	39.53 (12.31, 60.36)	9.74 (5.91, 25.99)	2.889	0.004
Mean	36.40 (27.20, 45.44)	21.60 (12.73, 33.77)	3.295	0.003

Data are expressed as median (quartile 1, quartile 3), and intergroup comparison was performed with Mann–Whitney U test.

BPT, Benign phyllode tumor; BMPT, borderline/malignant phyllode tumor; RLM, run-length matrix; GLCM, gray-level co-occurrence matrix; Z_GLevNonU, 135dr_GLevNonU, and 45dgr_ GLevNonU, gray-level non-uniformity calculated for Z-axis, 135-, and 45-degree directions, respectively; AngScMom, angular second moment; Perc.90%, percentile 90%.

The definition and formula of the above features were as follows:

*HIS Parameter 1: Perc.90%.* A percentile represents the value below which a percentage of observations is calculated.

*HIS Parameter 2: Mean*. Mean measures the average gray-level intensity within the VOI.


$$\text{Formula}:\Sigma k\frac{k^{*}g(k)}{\Sigma kg^{k}}$$

*HIS Parameter 3: Variance.* Variance is the mean of the squared distances of each intensity value from the mean value. This is a measure of the spread of the distribution about the mean. By definition, variance=σ^2^.


$$\text{Formula}:\frac{1}{N_{p}}\Sigma_{i=1}^{N_{p}}{\left(X(i)-\bar{X}\right)}^{2}$$

*GLCM Parameter: S(0,0,1)AngScMom and S(1,0,0)AngScMom.* AngScMom is a measure of image homogeneity. This feature obtains a high value when a gray-level distribution in the image is either constant or periodic.


$$\text{Formula}:\Sigma_{i,j}f{\left(i,j\right)}^{2}$$

*RLM Parameter: Z_GLevNonU, 45dgr_GLevNonU, and 135dr_GLevNonU.* GLevNonU measures the similarity of gray-level intensity values in the image, where a lower GLevNonU value correlates with a greater similarity in intensity values.


$$\text{Formula}:\frac{\Sigma_{i=1}^{N_{g}}{\left(\Sigma_{j=1}^{N_{r}}\;p(i,j|\theta )\right)}^{2}}{N_{r}(\theta )}$$

### Comparison of Conventional MRI Findings Between BPTs and BMPTs

**Figure 3:**
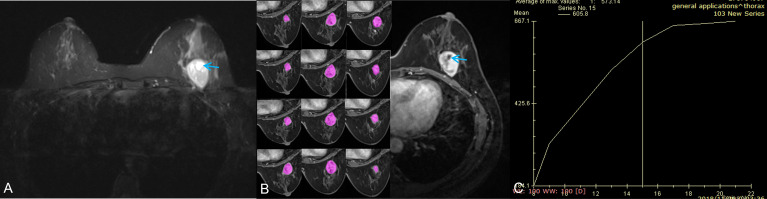
A 42-year-old woman with a benign phyllodes tumor. **(A)** Axial T2WI showed a heterogeneous mass in the left breast, with a regular wall of cystic area (red arrow). **(B)** Axial DCE-MRI showed the mass with heterogeneous enhancement and non-enhancement cystic area; the segmentation of VOI was shown on the left series images. **(C)** The time-intensity curve was type II (plateau pattern).

**Figure 4:**
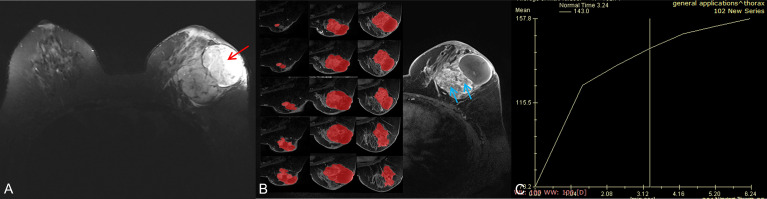
A 56-year-old woman with a borderline/malignant phyllodes tumor. **(A)** Axial T2WI showed a heterogeneous mass with a huge cystic cavity (red arrow) in the left breast. **(B)** Axial DCE-MRI showed the mass with heterogeneous enhancement and large numbers of unenhanced areas with irregular walls (blue arrow); the segmentation of VOI was shown on the left series images. **(C)** The time-intensity curve was type I (persistent pattern).

The conventional MRI findings of BPTs and BMPTs are summarized in **Table 3**. Between the two groups, except the cystic wall morphology, all the conventional MRI findings including tumor shape, cystic component, lobulation, margin, T2WI_FS_ signal, hyperintense on T2WI_FS_, hyperintense on T1WI, dark internal septation, enhancement signal, and TIC pattern showed no significant differences. The irregular cyst wall was more commonly seen in BMPTs (11/18, 61.1%) than in BPTs (5/22, 22.7%) (p = 0.014) (**Figures 3**, **4**).

**Table 3:** Conventional MRI findings between BPT and BMPT groups.

MRI findings	BPT (n = 26)	BMPT (n = 21)	p-value
Max diameter	4.58 ± 2.38	5.55 ± 2.18	0.159^a^
Shape			0.495^b^
Round	10 (38.5%)	7 (33.3%)	
Oval	9 (34.6%)	5 (23.8%)	
Irregular	7 (26.9%)	9 (42.9%)	
T2WI_FS_ signal			0.851^b^
Homogeneous	8 (30.8%)	7 (33.3%)	
Heterogeneous	18 (69.2%)	14 (66.7%)	
Hyperintense on T2WI_FS_			0.472^b^
Absent	3 (11.5%)	4 (19.0%)	
Present	23 (88.5%)	17 (81.0%)	
Hyperintense on T1WI			0.466^b^
Absent	22 (84.6%)	16 (76.2%)	
Present	4 (15.4%)	5 (23.8%)	
Cystic component			0.916^b^
Absent	4 (15.4%)	3 (14.3%)	
Present	22 (84.6%)	18 (85.7%)	0.014^b^
Regular wall	17 (77.3%)	7 (38.9%)	
Irregular wall	5 (22.7%)	11 (61.1%)	
Lobulation			0.933^b^
Absent	9 (34.6%)	7 (33.3%)	
Present	17 (65.4%)	14 (66.7%)	
Margin			0.774^b^
Regular	15 (57.7%)	11 (52.4%)	
Circumscribed	11 (42.3%)	10 (47.6%)	
Dark internal septation			0.900^b^
Absent	19 (73.1%)	15 (71.4%)	
Present	7 (26.9%)	6 (28.6%)	
Enhancement signal			0.137^b^
Homogeneous	14 (53.8%)	6 (28.6%)	
Heterogeneous	12 (46.2%)	15 (71.4%)	
TIC pattern			0.691^b^
Type I	8 (30.8%)	7 (33.3%)	
Type II	17 (65.4%)	12 (57.1%)	
Type III	1 (3.8%)	2 (9.5%)	

Quantitative variables are expressed as mean ± standard deviation. Qualitative variables are expressed as proportions. ^a^Data were performed with independent t-test. ^b^Data were performed with chi-square test. The level of significance for intergroup differences was set at p < 0.05.

BPT, benign phyllode tumor; BMPT, borderline/malignant phyllode tumor; T2WI_FS_, T2 weighted imaging with fat saturation; T1WI, T1 weighted imaging; TIC, time-intensity curve.

### ROC Analysis and Diagnostic Performance

**Figure 5:**
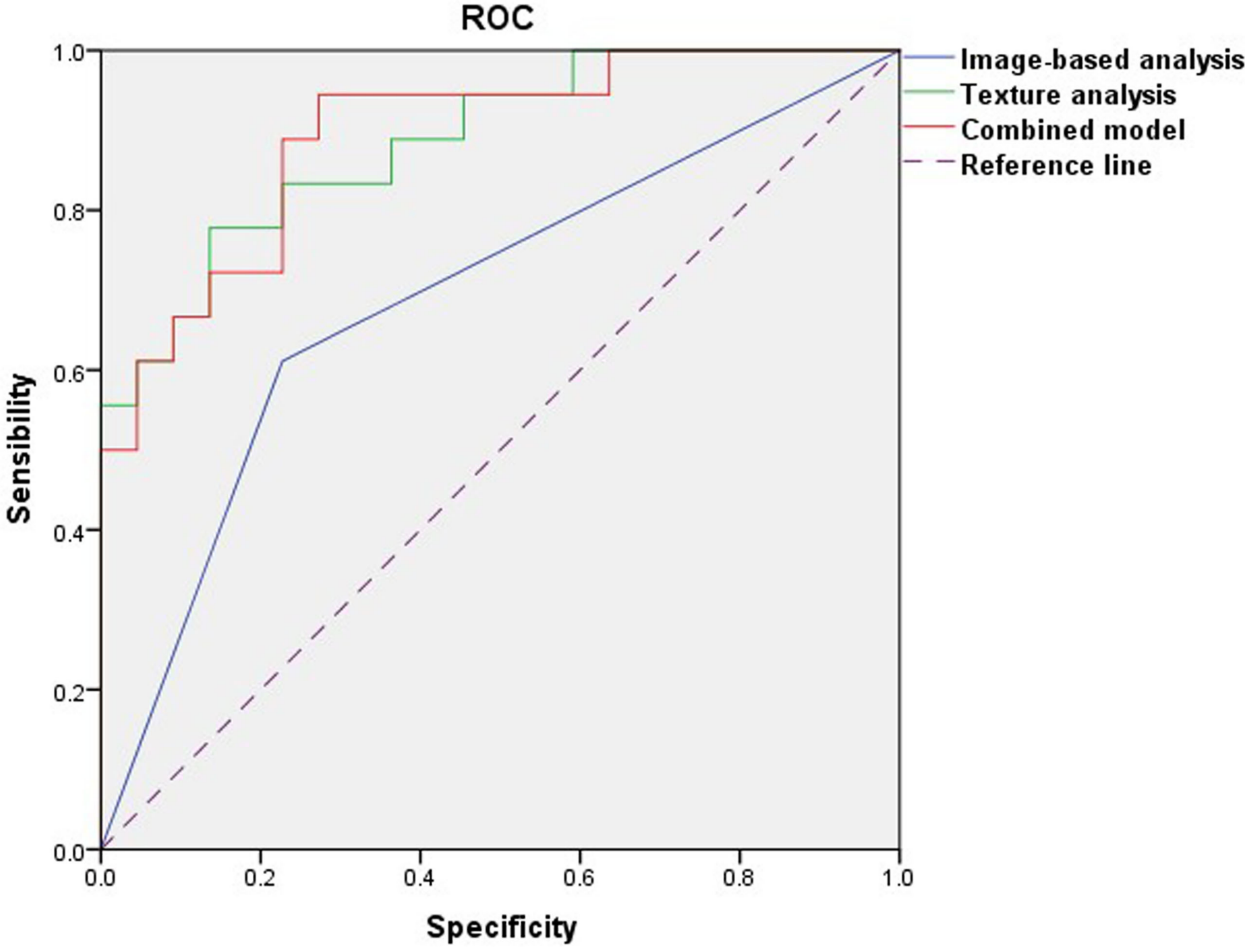
Receiver operating characteristic curves of image-based analysis, TA, and the combined model.

In the comparison of conventional MRI findings between BPTs and BMPTs, three parameters with p < 0.20 were obtained: they were the max diameter (p = 0.159), enhancement signal (p = 0.137), and cystic wall morphology (p = 0.014). Further multivariable logistic regression analysis found that the cystic wall morphology differed significantly between the two groups (p = 0.020) and was thus regarded as an independent variable. The final regression model achieved an AUC of 0.687 (sensitivity 61.1%, specificity 76.2%, and 95%CI, 0.518-0.825) (p = 0.014). Multivariate logistic regression analysis of eight texture features found that Z_GLevNonU, S(0,0,1)AngScMom, Perc.90%, variance, and mean differed significantly between the two groups (p = 0.029, 0.031, 0.004, 0.001, and 0.003, respectively) and were thus regarded as independent variables. The following equation was obtained: Logit(p) = 0.067×S(0,0,1)AngScMom + 0.001×Z_GLevNonU +1.944×Perc.90%–0.301×Variance–1.994×Mean–4.552. The model exhibited an AUC of 0.886 (95%CI, 0.760–0.960) (p < 0.0001), with a sensitivity of 85.7% and a specificity of 80.8%. The combined model showed an AUC of 0.894 (95%CI, 0.754–0.970) (p < 0.0001), with a sensitivity of 94.4% and a specificity of 76.2%. The Hosmer–Lemeshow test showed a good model fit for these models from image-based analysis, TA, and the combination of the two approaches (p = 1.000, 0.788, and 0.588, respectively) (**Table 4** and **Figure 5**). The Delong test showed that both the AUC of TA and the combined model were significantly higher than that of image-based analysis (p=0.010 and 0.003, respectively). However, no significant difference was found between TA and the combined model (p=0.893).

**Table 4:** ROC analysis of image-based analysis, texture analysis, and the combination analysis.

Logistic regression model	AUC	95% CI	Sensitivity	Specificity
Image-based analysis	0.687	0.518-0.825	0.611	0.762
Texture analysis	0.886	0.760-0.960	0.857	0.808
Combination analysis	0.894	0.754-0.970	0.944	0.762

ROC, receiver operating characteristic; AUC, area under receiver operator characteristic curve; CI, confidence interval.

## Discussion

The latest frontiers in medical image analysis have highlighted the implementation of computer vision principles and analytical techniques for quantifying and describing medical images. TA is a statistical method that can be used to characterize the gray-level signal intensity and its spatial variation within an image, capturing image patterns usually unrecognizable or indistinguishable to the human eye (10). Compared with conventional imaging methods, TA can provide objective and additional quantitative image information on lesions independent of the subjective judgment and experience of clinicians or radiologists, adding potential clinical value (18). Recently, TA has been widely used to evaluate tumor heterogeneity. Many studies also indicate that texture features are good predictors of breast tumor classification (10, 11, 19). In the present study, we attempted to evaluate the role of TA based on DCE-MR images in grading PTs. Our results suggested that TA based on DCE-MR images has potential in differentiating BPTs and BMPTs. We found that three histogram features, two GLCM features, and three RLM features were significantly different between BPTs and BMPTs. Moreover, TA or combined with imaging findings exhibited better diagnostic performance in differentiating BPTs and BMPTs than that from imaging analysis alone.

As a first-order texture, gray-level histogram analysis can be used to describe the distribution of pixel intensities within an image without considering the neighboring pixels. The mean value reflects the central trend and average level of grayscale, while the percentile provides the highest gray-level value that contains a given percentage of the pixels in the VOI. It has been suggested that the whole-lesion analysis of breast tumors instead of the single slice measurement may better depict the tissue heterogeneity (27). In this study, the mean gray value of BPTs was significantly greater than that of BMPTs, indicating that the average signal intensity of BPTs was higher than that of BMPTs on DCE-MR images. This result was consistent with our previous study (18), which showed that the average gray value obtained from a single slice was higher in BPTs than in BMPTs, though there was no intergroup difference. Additionally, we found that the variance and 90th percentile gray values in BPTs were also higher than those in BMPTs. Variance reflects the degree of dispersion between the gray values of an image, and the 90th percentile represents the pixels close to the highest gray values. The increased frequency indicates that the proportion of high signal pixels in the enhanced images of BPTs was higher than that in BMPTs. This indicates that there are more areas of higher brightness, or significant enhancement, in BPTs than in BMPTs. Therefore, the histogram analysis of the whole tumor has advantages in PT grading over that of a single slice.

The GLCM features are the most commonly extracted second-order texture features for MRI quantification, which were used to reflect the spatial relationship of pixel or voxel gray-level values in the image. The GLCM feature angular second moment (AngScMom) reflects the uniformity of the gray-level distribution, where a higher AngScMom value indicates a more homogenous image (10). Ma et al. (15) showed that the texture parameter SumAverage from DCE-MR images was significantly different in BPTs and BMPTs, which was identified as one of three significant predictors (Compactness, SumAverage, and Correlation) for PT grading. In our study, we also found that the values of S(0,0,1)AngScMom and S(1,0,0)AngScMom were significantly higher in BPTs than in BMPTs, which indicated that BPTs had a relatively homogeneous gray level and regular textures compared with BMPTs. RLM reflects the comprehensive information of the image grayscale concerning direction, adjacent interval, and variation amplitude. The RLM feature GLevNonU measures the similarity of gray-level intensity values in the image. The smaller the GLevNonU value is, the more times a certain gray level appears, and the more uniform the gray level of the corresponding image is. Many texture features are unstable in different reconstruction algorithms, while GLevNonU is one of the most repetitive radiomics features showing good stability. The GLevNonU value increases with the tumor heterogeneity, which is related to tumor invasion, treatment response, and prognosis (28). In this study, we found that the Z_GLevNonU, 45dgr_GLevNonU, and 135dr_GLevNonU values of the BMPTs were statistically larger than those of the BPTs, indicating that the gray-level distribution was more heterogeneous in BMPTs on DCE-MR images, compared with BPTs. Thus, combined with the pathological basis, we hypothesized that the significantly higher value of GLevNonU might be related to the greater heterogeneity caused by the more stromal atypia and cellular necrosis in BMPTs (29). This finding was similar to the results of a previous study of triple-negative breast cancer (TNBC) (30). By ultrasound (US) TA, patients with TNBC have a higher GLevNonU value than that in patients with non-TNBC, indicating that TNCB has higher heterogeneity and malignancy.

The BI-RADS lexicon has been widely used for more clear and concise communication of physicians and radiologists based on imaging findings to evaluate the classification and gradation of breast diseases (16). A previous study has described cysts and hemorrhage as typical signs of phyllodes breast tumors (8); however, our results showed that there was no difference between BPTs and BMPTs in the signal changes representing bleeding and cysts on T1W or T2W_FS_ images. According to the BI-RADS diagnostic criteria of breast MRI (16), one of the descriptions of the nature of a mass lesion is the internal enhancement characteristics, which can be divided into homogeneous, heterogeneous, rim enhancement, etc. In this study, the irregular heterogeneous enhancement was more common in BMPTs; however, consistent with previous studies (6, 8), there were no significant differences in the enhancement type and the TIC type between BPTs and BMPTs. Tumor size was considered to be an important factor for PTs’ biological behavior (6). Our results showed that the malignancy rate increased with increasing tumor size. This finding reflects the high proliferative activity of BMPTs, though there was no significant difference among the PTs. Well-defined margins with a round or lobulated shape and a septate inner structure have been described as characteristic morphologic signs of PTs (15). However, our study showed no significant difference among BPTs and BMPTs in terms of lesion shape. The cystic component was found in 22 cases of BPTs (84.6%) and 18 cases of BMPTs (85.7%), with no intergroup difference. Interestingly, however, we found that the irregular cyst wall was more commonly seen in BMPTs (11/18, 61.1%) than in BPTs (5/22, 22.7%), with a significant intergroup difference. Multivariable logistic regression analysis further showed that irregular cystic walls could be an independent factor for differentiating BPTs from BMPTs. Therefore, in this study, the irregular cystic wall could be used as a valuable imaging label for differentiating BPTs from BMPTs.

In this study, ROC analysis was adopted to evaluate the diagnostic efficacy for the models from image-based analysis, TA, and a combination of the two approaches in differentiating BPTs from BMPTs. The results indicated that the diagnostic performance of the TA model or the combination model was greater than that achieved with image-based analysis alone (AUC: 0.894 vs 0.886 vs 0.687), even though there was no difference between the TA model and the combination model. Therefore, compared with conventional imaging findings based on human visual analysis, TA or combined with imaging findings has the potential in improving the differential diagnosis ability between BPTs and BMPTs, which is consistent with the result of a previous study by Cui et al. (31), who found that combining mammography findings and texture features can provide optimal predictions in the classification of PTs in mammography.

We acknowledge the following limitations in our study. Firstly, as a retrospective study, the limited number of samples, especially for the malignant PTs, may lead to inherent variations and selected bias and therefore impact the accuracy of the result. Secondly, as a single-center retrospective study, the results needed to be externally validated through a multicenter study. Thirdly, texture features are only obtained from the DCE-MR image; however, it is not ruled out that more meaningful quantitative features derived from other sequences, such as T2WI, DWI, will produce more diagnostic performance. Fourthly, manual VOI segmentation led to inevitable measurement errors; thus, the next step is to resort to semiautomatic or artificial intelligence tools that can accurately recognize these lesions. Finally, although the texture features provided a quantitative method of classifying breast lesions, we have to admit that the direct biological interpretation of texture features remains largely uncertain.

## Conclusions

Our results suggest that the TA based on DCE-MR images has the potential to differentiate BPTs and BMPTs. Compared with traditional imaging analysis, TA or combined with imaging findings yielded better diagnosis performance for PT grading. Considering that it is a relatively small sample size and single-center study, future validation studies with multiple centers are needed to verify its clinical feasibility.

## Data Availability Statement

The data analyzed in this study are subject to the following licenses/restrictions: the data supporting the conclusions of this study are available upon request for researchers who met the criteria for access to confidential data. Requests to access these datasets should be directed to X-Li, (18225023834@163.com).

## Ethics Statement

This study was approved by the institutional ethics committee of Da-ping Hospital of Army Medical University [Ratification NO: 2019(160)]. The ethics committee waived the requirement of written informed consent for participation.

## Author Contributions

JF and X-Li conceived and designed the study. HS collected the MRI data. CZ and X-Luo analyzed MRI data. PZ provided pathology results. CC performed figure and table preparation. HL performed statistics. X-Li and HG wrote the manuscript. JF and YW edited the manuscript. All authors revised the manuscript, read, and approved the submitted version.

## Funding

This work was supported by the Fund of National Natural Science Foundation of China (81671943), Chongqing Health Commission Fund (2022WSJK029), Chongqing Clinical Research Centre of Imaging and Nuclear Medicine (CSTC2015YFPT-gcjsyjzx0175), and Central government guide of the development of local science and technology special fund (YDZX20175000004270).

## Conflict of Interest

Author HL was employed by GE Healthcare.

The remaining authors declare that the research was conducted in the absence of any commercial or financial relationships that could be construed as a potential conflict of interest.

## Publisher’s Note

All claims expressed in this article are solely those of the authors and do not necessarily represent those of their affiliated organizations, or those of the publisher, the editors and the reviewers. Any product that may be evaluated in this article, or claim that may be made by its manufacturer, is not guaranteed or endorsed by the publisher.

## Abbreviations

BPTs, benign phyllodes tumors; BMPTs, borderline/malignant phyllodes tumors; AUC, area under the receiver operating characteristic curve; HIS, gray-level histogram; GLCM, gray-level co-occurrence matrix; RLM, run-length matrix; ROC, receiver operating characteristic; VOI, volume of interest; GLevNonU, gray-level non-uniformity; TA, texture analysis.
